# Risk of depression in family caregivers: unintended consequence of COVID-19

**DOI:** 10.1192/bjo.2020.99

**Published:** 2020-10-12

**Authors:** Stephen Gallagher, Mark A. Wetherell

**Affiliations:** Department of Psychology, Centre for Social Issues Research, Study of Anxiety, Stress and Health Laboratory and Health Research Institute, University of Limerick, Ireland; Stress Research Group, Department of Psychology, Northumbria University Newcastle, UK

**Keywords:** Caregivers, COVID-19, depression, isolation, loneliness

## Abstract

**Background:**

Coronavirus disease 2019 (COVID-19) is likely to exacerbate the symptoms of poor mental health in family caregivers.

**Aims:**

To investigate whether rates of depressive symptomatology increased in caregivers during COVID-19 and whether the unintended consequences of health protective measures, i.e., social isolation, exacerbated this risk. Another aim was to see if caregivers accessed any online/phone psychological support during COVID.

**Method:**

Data (1349 caregivers; 6178 non-caregivers) was extracted from Understanding Society, a UK population-level data-set. The General Health Questionnaire cut-off scores identified those who are likely to have depression.

**Results:**

After adjustment for confounding caregivers had a higher risk of having depressive symptoms compared with non-caregivers, odds ratio (OR) = 1.22 (95% CI 1.05–1.40, *P* = 0.008) evidenced by higher levels of depression pre-COVID-19 (16.7% caregivers *v.* 12.1% non-caregivers) and during the COVID-19 pandemic (21.6% caregivers *v.* 17.9% non-caregivers), respectively. Further, higher levels of loneliness increased the risk of depression symptoms almost four-fold in caregivers, OR = 3.85 (95% 95% CI 3.08–4.85, *P* < 0.001), whereas accessing therapy attenuated the risk of depression (43%). A total of 60% of caregivers with depression symptoms reported not accessing any therapeutic support (for example online or face to face) during the COVID-19 pandemic.

**Conclusions:**

COVID-19 has had a negative impact on family caregivers’ mental health with loneliness a significant contributor to depressive symptomatology. However, despite these detriments in mental health, the majority of caregivers do not access any online or phone psychiatric support. Finally, psychiatric services and healthcare professionals should aim to focus on reducing feelings of loneliness to support at-risk caregivers.

## Background

The coronavirus disease 2019 (COVID-19) pandemic has obvious widespread effects on physical health; however, as the pandemic continues, there is also an increasing and significant impact upon mental health.^1^ For example, a recent-meta-analysis has found evidence of higher rates of depression in front-line workers during the pandemic relative to non-pandemic population norms.^2^ Wide-scale public health interventions have been implemented internationally to contain the COVID-19 outbreak (for example school and business closures, physical distancing measures, quarantine, shielding or cocooning of at-risk individuals (shielding/cocooning are concepts used in the UK and Ireland to describe social isolation procedure instructions to protect the medically vulnerable from COVID-19)), alongside curtailment of many health and social non-emergency services. However, these inventions are likely to have unintended consequences, especially for those who are vulnerable psychiatrically. In response, a recent consortium of psychiatrists and psychologists has strongly advocated for concerted efforts to research the impact of the pandemic on those groups that are most vulnerable.^3^

Family caregivers (i.e. those that provide long-standing care for a family member) are an integral part of the care of the medically and psychiatrically vulnerable. The caregiver experience entails continuous demands (for example provision of personal, health and social care to relatives) that can be extensive both physically and emotionally, leading to a significant stress burden.^4^ While some caregivers cope, others do not and are at increased risk of psychological morbidity such as the development of depression.^4,5^

As such, family caregivers themselves have been identified as a psychiatrically vulnerable group,^3,5^ especially when their own needs are not met.^3^ This is an increasingly likely scenario during the COVID-19 pandemic where strict limitations on movement; curtailment of non-emergency medical treatment; and changes to delivery (such as online or phone supports), or cessation of health and social care services is putting increasing pressure on caregivers to deliver care beyond their usual caring responsibility.^3,6,7^ Moreover, changes to policy around redeployment of health and social care staff, postponing of patient treatments and services has directly had an impact on family carers (for example curtailment of respite services for their loved ones, cancelled out-patient visits or follow-up visits for disease and health monitoring) which are likely to be additional stressors.^7^

Under normal conditions, caregivers routinely report having minimal opportunity for respite, little time for self-care and increased social isolation, which together in combination with the current pandemic are likely to increase their risk of depression. With restrictions likely to be extended into the future, in some guise or another, this risk will increase; studying the impact of COVID-19 on caregivers is therefore clearly warranted. In addition, the well-being of the caregiver goes hand-in-hand with those for whom they provide care. If the ability to provide care and support to their family members is hindered because of their own health crisis research has identified a greater risk of institutionalisation of loved ones, as well as additional economic, health and social costs.^8^ It is well-documented that stress can increase vulnerability to physical disease,^9^ making caregivers at potentially greater risk of developing COVID-19. It is therefore important to identify family caregivers who are experiencing a deterioration in their mental health, and understand the factors that contribute to their increased risk (i.e. social isolation, having to provide extra care), as these may reduce their vulnerability and inform future practice and treatment.^3^

## Aims

Thus, the present longitudinal case–control population-level study presented in this paper explores changes in levels of depression, a common marker of carer-related psychological morbidity, and other potential contributory factors (i.e. loneliness), in family caregivers and non-caregivers assessed pre-COVID-19 and during the COVID-19 pandemic. We hypothesised that (a) family caregivers would have a higher rate of depressive symptomatology at both time points relative to non-caregivers; (b) that family caregivers who feel more isolated/lonely and who are caring more during the pandemic will have higher risk of being depressed. Finally, given that access to treatment may have been affected or been altered (for example online/phone) in response to COVID-19, we also examined if caregivers with and without depression symptoms were availing of psychiatric or psychological support during the COVID-19 pandemic.

## Method

### Study design and participants

A longitudinal study design was employed by using two waves of the Understanding Society/ UK Household Longitudinal Study. This is a longitudinal survey of approximately 40 000 households in the UK; data were extracted from Wave 9, which was pre-COVID-19 (2017–2019),^10^ and from the specially commissioned Understanding Society COVID-19 Wave (May 2020).^11^ All participants from the Understanding Society Study gave informed consent and ethics were obtained by the University of Essex, UK from National Research Ethics Service Oxfordshire REC A (08/H0604/124).

Caregiving was ascertained by asking participants: ‘Is there anyone in your own home who is sick, disabled or elderly whom you look after or give special help to’ which had a yes/no format. A similar question was asked for those caring outside the home. These approaches have been used elsewhere to capture caregiver samples,^12^ and those answering yes, to both were pooled as one homogeneous caregiver group.^13^ For analysis, caregivers and non-caregivers had to have participated in both waves. The final sample was *n* = 7527 (*n* = 1349 caregivers). Relationship, ethnicity, and job status were dichotomised (for example married/partnered versus single/divorced/widowed; White versus Black and minority ethnic; employed versus unemployed/retired). Similarly, living arrangements were ascertained by asking if they lived with a partner (yes/no), and how many individuals in several age brackets were living there: ages 0–4, 5–15, 16–18, 19–69, and ≥70 years old . These were totalled and recoded into: 0, living alone and 1, living with others (see Table 1 for group characteristics).

**Table 1:** Sociodemographics, health and outcome variables across caregivers groups

Variable	Non-carers (*n* = 6178)	Carers (*n* = 1349)	*F* (d.f.)	*χ^2^*(d.f.)	*P*
Age, years: mean (s.d.)	47.5 (17.70)	52.8 (14.79)	142.56 (1,9768)		<0.001
Married/partnered, %	74.2	79.5		16.82 (1)	<0.001
Gender, women: %	51.3	61.5		21.00 (1)	<0.001
Ethnicity, White: %	92.6	93.6		2.10 (1)	0.14
Income, monthly £: mean (s.d.)	1700 (1785)	1635 (1585)	2.03 (1,9768)		0.15
Employed, no: %	35.9	27.2		50.89 (1)	<0.001
Disability, yes: %	31.2	43.9		100.38 (1)	<0.001
Living alone, yes: %	2.0	3.4		100.38 (1)	<0.001
Pre-COVID-19 lonely, often: %	7.5	8.0		11.90 (2)	<0.001
COVID-19 lonely, often: %	7.1	8.2		2.73 (2)	0.25
Accessed therapy, no: %	42.1	59.7		375.5 (2)	<0.001
Pre-COVID-19 depression, yes: %	12.1	16.7		26.68 (1)	<0.001
COVID-19 depression, yes: %	17.9	21.6		12.45 (1)	<0.001

### Outcomes

Depression symptomatology was our primary outcome and captured in both waves of the Understanding Society Study by the 12-item General Health Questionnaire.^14^ Items (for example unhappy or depressed) are scored as 1, not at all; 2, no more than usual; 3, rather more than usual; 4, much more than usual. Responses of 1 or 2 are scored as 0, and responses of 3 or 4 are scored as 1. A total score ≥6 is specific and sensitive at identifying those with or without a depressive disorder.^15^

### Access to therapy during COVID-19

Access to therapy was a secondary aim and was assessed in the COVID-19 Understanding Society study by asking: ‘In the last month, have you accessed counselling or talking therapy?’, response categories were: 1, yes, in person; 2, yes, by telephone or online; 3, yes, group sessions; 4, no; and 5, not required.

### Loneliness

Loneliness at both time points in both waves was assessed by a single item: ‘In the last 4 weeks, how often did you feel lonely?’ with three responses, 1, hardly ever or never; 2, sometimes; 3, often.

### Extra caring

More caring responsibility during COVID-19 was assessed by the question ‘How has the help and support you receive from family, friends or neighbours who do not live in the same house/flat as you changed?; responses were: 1, there has been no change; 2, I receive more help from some people who previously helped me; 3, I receive less help from some people who previously helped me; 4, I currently receive help from family, friends or neighbours who did not previously help me; 5, Other.

### Statistical analysis

Weights were calculated to provide a representative national sample, taking into account survey design and non-response. For more information on the weighting system, see the main Understanding Society COVID-19 report.^11^ No outliers were observed and data was normally distributed. There was a low response rate (*n* = 2) for the access to therapy response face-to-face therapy (1 who was depressed and 1 who was not depressed) and *n* = 1 (who was depressed) for group sessions. Thus, we pooled these with the online/phone group response as all respondents had access to therapy to produce three ‘access to therapy’ categories: 1, accessed online or phone treatment; 2, no access; and 3 did not require treatment.

Tests of differences were used to examine group differences on sociodemographic, health and outcome variables. Logistic regression was used to examine the predictors of risk of likely depression in caregivers relative to non-caregivers. In this analysis, non-caregivers and those without depression were both dummy coded as 0, whereas caregivers and those with depression symptoms were coded at 1. Confounding variables (i.e., health and sociodemographics, pre-existing depression and loneliness) were entered in step one of the model and caregiver groups (non-caregivers versus caregivers) entered at step two. This was followed by another logistic regression analysis for caregivers only, to examine the predictors of likely depression risk with the same confounding factors entered in step one, pre-COVID depression symptoms and loneliness at step two, and current COVID-19-related loneliness, access to treatment and more or less caring during the crisis in step three. Odds ratio (OR) is the effect size.

## Results

As can be seen in Table 1, caregivers were slightly older, more likely to be married/partnered, be women, be unemployed/retired during COVID-19, live alone and have a health condition/disability in comparison to non-caregivers. As expected, rates of depression symptoms increased during the pandemic for both groups. However, caregivers had higher rates of likely depression (16.7%, 21.6%) than non-carers (12.1%, 17.9%), at pre-COVID-19 and during COVID-19 time points, respectively.

In logistic regression, after controlling for confounding factors (see Table 1 for group differences) in step one, caregivers had a 21% greater risk of being depressed compared with non-caregivers; OR = 1.22 (95% CI 1.05–1.40, *P* = 0.008).

In within-caregiver group analysis (see Table 2), after controlling for confounding variable in step one and step two, we found that the key predictor of this excess risk was current loneliness such that those who felt lonelier during the current crisis had an almost four-fold risk of depression, OR = 3.85 (95% CI 3.08–4.85, *P* < 0.001).

**Table 2:** Hierarchical logistic regression sociodemographic, health, loneliness, caring more/less and access to therapy predicting family caregiver depression

Variables	*B*	Odds ratio	*P*	95% CI lower	95% CI upper
Step 1					
Age	−0.01	0.99	0.23	0.97	1.06
Partnered	−0.01	0.70	0.94	0.61	1.58
Gender	0.84	2.32	0.001	1.54	3.49
Ethnicity	0.77	2.26	0.033	1.06	4.29
Income	0.00	1.00	0.12	1.00	1.00
Employment	−0.01	0.99	0.91	0.98	1.03
Living alone	0.62	1.85	0.11	0.86	4.00
Disability	−0.29	0.75	0.14	0.51	1.07
Step 2					
Pre-COVID depression	1.05	2.76	0.001	1.83	4.15
Pre-COVID loneliness	0.05	1.09	0.53	0.82	1.46
Step 3					
Access to therapy	−0.56	0.57	0.001	0.40	0.80
More or less caring duties during the COVID-19 outbreak	0.09	1.09	0.68	0.70	0.168
COVID-19 loneliness	1.35	3.85	<0.001	3.08	4.85

**Fig. 1:**
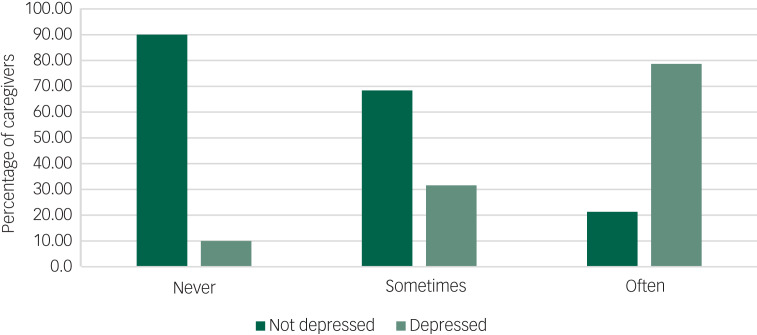
Depression and feelings of loneliness (‘never’, ‘sometimes’ or ‘often’) during the COVID-19 pandemic in caregivers.

As can be seen in Fig. 1, almost 80% of those who reported being lonely ‘sometimes’ were depressed, whereas 90% of caregivers who were not depressed reported never being lonely. Access to psychological supports reduced depressive risk by 43% (see Table 2).

**Fig. 2:**
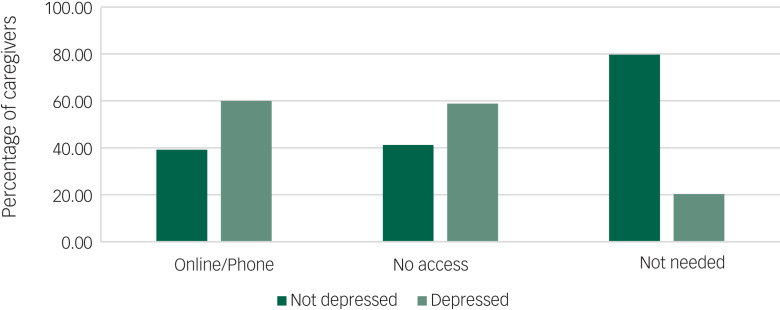
Depression and access to psychological therapy (‘online/phone’, ‘no access’, ‘not needed’) during the COVID-19 pandemic in caregivers.

Finally, we explored how access to treatment varied across caregivers with and without likely depression. As can be seen in Fig. 2, 60% of carers with likely depression said they did not access any psychological supports, and 20% of caregivers with depression symptomatology reported ‘no need for support’ (χ^2^(2) = 78.63, *P* < 0.001)

## Discussion

The present study demonstrates that levels of depression symptomatology have increased in a large sample of UK citizens during the COVID-19 pandemic. Additionally, and in line with previous research, caregivers report greater levels of symptoms of depression compared with non-caregivers and this is evident both pre-COVID and during the COVID-19 pandemic. Furthermore, current levels of loneliness were a significant predictor of depression risk while access to psychological support attenuated the risk. However, it is worth noting that a large proportion of caregivers with depression symptoms felt they did not need psychotherapy support.

It is likely that the public health restrictions implemented to protect people from COVID-19 are having unintended consequences,^3,7^ as evidenced by the fact that current feelings of loneliness and not previous loneliness, exacerbated caregivers’ likely depression risk. Moreover, this was evident irrespective of whether caregivers lived alone or not. Further, our findings also showed that although 20% of caregivers who were categorised with likely depression felt they did not need access to therapy, a large percentage (60%) of caregivers with depression symptoms reported having no psychological support during the pandemic.

Our findings align with recent observations showing that risk of likely depression has increased during the current crisis.^2^ In the present study, we have demonstrated that this risk is exacerbated in family caregivers, by comparing levels of depression symptoms from pre-COVID-19 to levels experienced during the current crisis. Levels of depression symptoms as indexed using a ≥6 on the GHQ^15^ to identity those with depression, increase by 5% in family caregivers during this period. It is worth noting that there are over 6.5 million family caregivers in the UK,^16^ and taking this as our population estimate, this increase of 5% equates to an additional 325 000 family caregivers who are likely to have depression.

Thus, the recent call to investigate the negative effects of COVID-19 on those with existing psychiatric vulnerabilities was warranted and confirmed by the findings of this study.^3^ More importantly, we have identified the effects of isolation, a likely consequence of restrictions aimed at reducing the spread of COVID-19, as a potential contributor to increased depression. In fact, it was loneliness experienced during the pandemic and not previous levels of loneliness that was a significant contributor, with caregivers who experienced greater levels of loneliness having an almost four-fold increased risk of depression.

Further, it is interesting to note that changes to their caring duties were not predictive of depression symptoms, and it does suggest that it is the unintended psychological consequences of COVID-19 restrictions, i.e. loneliness, and not the physical demands of having to care more, that are contributing to levels of depression. Despite increases in levels of depression symptoms during the COVID-19 pandemic, and the benefits of accessing therapeutic treatments, a significant proportion of caregivers reported no access to psychological support during this period. This suggests that for many psychologically vulnerable individuals, support is either not available or not being sought.

### Limitations

This study has several strengths, notably a longitudinal design and the use of a validated screening tool for the assessment of depression symptoms in population research. In response to recent calls, it also assesses an at-risk group and identifies potential contributory factors linked to depression. However, these findings should be considered in light of some limitations.

First, we do not have the details of the type of care-recipients’ illness/disability type which may confer an additional risk and differences in the caregiver experience and associated care burden (for example Alzheimer's disease and cancer). Second, there may be other unmeasured variables that may also explain risk of likely depression (such as worry and anxiety), and as can be seen in Fig. 2, some 40% of caregivers were not categorised as depressed but were availing of treatments, and as such may have had another mental health condition.

Third, although caregiving was predictive of depression at both time points, we cannot infer causality. Increased psychiatric symptomatology and rates of common mental disorders in caregivers could also reflect shared biological vulnerabilities with their care-recipient relatives. Although plausible, there is convincing evidence that it is the caregiver role itself that drives increases in psychological morbidity such as depression,^5^ Fourth, our sample is predominantly White, middle-aged and women, and generalising to other caregivers should be undertaken with caution even though women are more likely to be caregivers; the peak age of caring is 50–64 years of age and Black, Asian and ethnic minorities caregivers are more likely to be younger.^17^

Finally, depression status was derived through a self-report scale rather than psychiatric interview. Nonetheless, we used a widely used scale, which has intrinsic value as an indicator of psychological distress, particularly in population studies such as this,^15,18^ Also, a lack of formal diagnosis of depression may explain why a large proportion of the caregivers were not accessing any psychological support resources. That is, despite reporting high levels of depression symptoms using a questionnaire, they may not have identified the need for accessing support that would typically be made available following a formal diagnosis. Moreover, we do not know whether individuals were accessing support services before the COVID-19 pandemic.

### Implications

Notwithstanding these limitations, this study demonstrates that caregivers are at increased risk of likely depression, and that feelings of isolation may increase this risk. Current and future restrictions related to social distancing will exacerbate this issue, and as such, caregivers are an especially vulnerable group during the COVID-19 pandemic. Given the well-established consequences of caregiving stress on physical health^4^ and vulnerability to viral infection,^19^ caregivers may be at increased risk of contracting COVID-19, which would have detrimental effects on their ability to provide care and implications for institutionalisation of relatives as well as additional health and social care costs.

The identification of caregivers as an at-risk group can therefore act as a prompt for psychiatric and mental health services to better understand the causes and consequences of psychological morbidity in this group. For example, this study has identified the potential role of perceived isolation as a risk factor for depression. As the pandemic continues caregivers may become greater users of mental health services, and the identification of risk factors may provide targets for intervention and treatment. Of significant note; however, is the observation that many of the caregivers in this study did not access any support services, despite their levels of depression symptoms. This is rather worrying as there are studies showing that family caregivers contemplate suicide more than non-caregivers and depression has been identified as one of the risk factors.^17,19^ Thus, these particular findings have implications for current practice, and how to tailor or address the needs of this vulnerable group and ensure that appropriate treatments and support services are accessible as quickly as possible, which could include medication^20^ and/or psychosocial support for depression including third-sector support online support groups for reducing isolation.^21,22^

Attempts to reduce the burden of caregiving has clear implications for the psychological and physical health of the carer, the well-being of the care recipient, and potential societal and financial costs. This is not a new challenge and there are ongoing endeavours to address this need. However, given the potential role of feelings of isolation as a risk factor, the ongoing impact of the COVID-19 pandemic raises specific issues for caregivers and thus, efforts to alleviate this risk are needed.^20^

## Data Availability

The data that support the findings of this study are openly available in Understanding Society: COVID-19 Study, 2020 at http://doi.org/10.5255/UKDA-SN-8644-1 and Wave 9 at http://doi.org/10.5255/UKDA-SN-6669-11
